# The Midnight Bite: The Quest to Combat the Emergence of Screwworm

**DOI:** 10.4269/ajtmh.26-0195

**Published:** 2026-06-02

**Authors:** Monica R. A. Pachar Flores

**Affiliations:** Infectious Disease Service, Hospital Santo Tomas, Panama City, Panama; Infectious Diseases, Instituto Oncologico Nacional, Panama City, Panama

Doña “Carmen,” a 50-year-old woman, sat across from me in my infectious disease and tropical medicine clinic at Hospital Santo Tomas in Panama City, Panama. She had been receiving treatment for cutaneous leishmaniasis, and I was happy to see her lesion steadily improving. Although I took heart in her progress, a shadow of preoccupation hung over Carmen.

“Doctor,” she said, voice trembling, “I’m not sure what to do. My cattle have deep, rotten wounds full of maggots. Some wounds are so bad that their horns have rotted and fallen off. I am worried that whatever this illness is will affect not only the animals but my community as well.” Dona Carmen was originally from El Silencio, a rural village located in Rio Indio District and part of Coclé Province, which is a mountainous zone in the central cordillera of West Panama. She had moved to Panama City many years ago but had maintained her family ranch, which had been raising cattle for generations.

Carmen and her family were familiar with infectious diseases that affected their cattle herds. Cases of “blackleg” and bovine paralytic rabies from vampire bat bites were known threats. However, something was different this year. Carmen noted that every time a vampire bat bit the cattle, if the wound was not cleaned promptly, it would develop “gusaneras” or maggot infestation. She showed me photos of the wounds and worms. The pictures showed wounds heavily infected with cream-colored larvae densely clustered within the ulcerated cavity. The larvae had circumferential bands of cuticular spines along body segments and a tapered anterior end with strong buccal hooks. In some, dark dorsal tracheal trunks and posterior spiracles with incomplete peritreme were visible through the posterior segments. The maggots were morphologically consistent with *Cochlomya hominivorax*, larvae of the New World screwworm (NWS) fly.

New World screwworm has been recognized in the Americas since the nineteenth century, but Charles Coquerel first described its larval stages causing myiasis in humans and animals in French Guiana in 1885. During the construction of the Panama Canal, veterinary records and reports from the Canal Zone were also documented. Research and control efforts targeting NWS in Panama continued in the following decades, including the introduction of the sterile insect technique in the 1960s. In 1994, the Panama–US Commission for Eradication and Prevention of Screwworm was established.

The Panama–US Commission for Eradication and Prevention of Screwworm is a binational program to control and prevent reinvasion of *C. hominivorax* that is operated by the US Department of Agriculture Animal and Plant Health Inspection Service in the United States and Ministerio de Desarrollo Agropecuario (Ministry of Agricultural Development) in Panama. Its primary mission is to maintain a biological barrier in eastern Panama (Darien Province) to prevent spread northward from endemic regions of South America. The program uses sterile fly production, aerial releases of sterile males, veterinary surveillance of livestock, and rapid treatment and containment of infestations. However, after years of success, since 2023, cattle cases in Panama have dramatically increased, leading to the rapid spread of NWS throughout Central America and Mexico. Currently, the high-risk zone for NWS resides in eastern Panama in Darien Province.

But, El Silencio is far from the Panamanian NWS hot zone. Unfortunately, it is also far from primary care centers and even farther from academic centers that monitor disease outbreaks and new infectious pathogens. Because of the lack of surveillance in the area and the lack of connection to health care facilities, Carmen noted that in her cattle, small wounds became large, debilitating wounds owing to maggot infestation. Frustrated by delays and limited access to care, Carmen hoped this visit would be more than just a follow-up for her cutaneous leishmaniasis but also, a chance to learn how to protect her cattle and the other animals in her community from the maggot infestation. She feared that it could also be transmitted to humans.

So, we talked. As a first step, I sketched a simple diagram of bat bites and the NWS worm life cycle (bat, bat bite, wound, larvae deposited into the wound, and festering of the wound) to illustrate how vampire bats feed at night, creating an entry point for flies to deposit screwworm larvae. Doña Carmen paused, then asked softly, “So are the bats causing these infestations?” I explained to her that it was not that straightforward. The bats provide the break in the skin that allows the flies to lay their eggs in the flesh, which then hatch and cause further tissue destruction. But, the bats alone were not the culprit.

Drawing on her years of experience on the family farm, Doña Carmen remembered the impact of vampire bat bites on her herd. She had seen cows stagger and salivate excessively, requiring culling because of rabies infection. Her family had historically trapped bats, applying poison to their feet so that they would carry it back to their cave and die, to prevent the spread of bovine rabies. More recently, the family recognized the role of preventative care and vaccination to prevent rabies in cattle and humans while safeguarding the bats’ integral role in local ecosystems. The question now was what to do to prevent the spread of screwworm in the village without affecting the bat population.

Second, we devised a plan. I told her that all wounds, including vampire bites, must be promptly inspected and treated to prevent infestation. Wounds should be cleaned with water and antiseptic as fresh wounds attract flies that lay eggs. After cleaning, use topical insecticidal sprays or powders, like violeta de genciana or sulfadiazina de plata, to prevent fly oviposition and aid healing. For prevention, drugs like doramectin or ivermectin can be used. Spray baths with insecticidal and repellent products and pour-on formulations containing cypermethrin or fipronil applied along the dorsal midline are also recommended.

Determined to act, she left my clinic with a plan. After one month, she came back to my clinic for a follow-up on her cutaneous leishmaniasis and an anticipated El Silencio update. Using the recommendations from our conversation, she connected with her brother in El Silencio and developed their plan against NWS. Together, with medical supplies in hand, they completed an 8-hour journey: 3 hours by car, then horseback, and finally, on foot carrying a cooler of antiseptics, topical insecticides, antibiotics, and rabies vaccines to El Silencio. They distributed wound care supplies house by house. Doña Carmen and her family spent several days vaccinating cattle against rabies, recording screwworm larvae sightings, and notifying local agricultural authorities and the Panama–US Commission for Eradication and Prevention of Screwworm. She demonstrated wound-cleansing techniques that she had learned from me to her kin so that they could continue proper wound care when she was gone. She was even able to send before-and-after treatment photos by mobile phone so that I could provide medical guidance on difficult cases.

Dona Carmen became a medical liaison in her community and an important contributor in combating the rise of NWS in rural Panama. She often speaks about the need for reliable communication systems and stronger local prevention and control programs for neglected tropical diseases at the community level, knowing firsthand the impact that preparation and communication can have in preventing emerging infectious diseases.

In El Silencio, a woman delivers vaccines and treatments on horseback and on foot not just to save cattle or improve her family’s life but to restore balance within her community. Tropical medicine, even when practiced from a tertiary hospital in the capital, must cross borders between disciplines, species, and sectors.

Doña Carmen’s journey reminds us that human health and animal health are inextricably linked and that the stories that we hear in the clinic may be the first signs of broader ecological distress. If we take these signals seriously, we have a small window to reduce the impact of emerging infectious diseases across our global community.

